# Sex differences in COVID‐19 symptom severity and trajectories among ambulatory adults

**DOI:** 10.1111/irv.13235

**Published:** 2023-12-19

**Authors:** Samuel P. Massion, Amanda C. Howa, Yuwei Zhu, Ahra Kim, Natasha Halasa, James Chappell, Trey McGonigle, Alexandra M. Mellis, Jessica E. Deyoe, Carrie Reed, Melissa A. Rolfes, H. Keipp Talbot, Carlos G. Grijalva

**Affiliations:** ^1^ Department of Health Policy Vanderbilt University Medical Center Nashville Tennessee USA; ^2^ School of Medicine Vanderbilt University Nashville Tennessee USA; ^3^ Department of Biostatistics Vanderbilt University Medical Center Nashville Tennessee USA; ^4^ Department of Pediatrics Vanderbilt University Medical Center Nashville Tennessee USA; ^5^ Influenza Division Centers for Disease Control and Prevention Atlanta Georgia USA; ^6^ Department of Medicine Vanderbilt University Medical Center Nashville Tennessee USA; ^7^ Department of Biomedical Informatics Vanderbilt University Medical Center Nashville Tennessee USA

**Keywords:** adult, ambulatory, COVID‐19, outpatient, sex differences, symptom severity

## Abstract

**Background:**

The ongoing COVID‐19 pandemic has led to hundreds of millions of infections worldwide. Although differences in COVID‐19 hospitalization rates between males and females have been described, many infections in the general population have been mild, and the severity of symptoms during the course of COVID‐19 in non‐hospitalized males and females is not well understood.

**Methods:**

We conducted a case‐ascertained study to examine household transmission of SARS‐CoV‐2 infections in Nashville, Tennessee, between April 2020 and April 2021. Among enrolled ambulatory adult participants with laboratory‐confirmed SARS‐CoV‐2 infections, we assessed the presence and severity of symptoms (total, systemic, and respiratory) daily using a symptoms severity questionnaire, from illness onset and throughout the 2‐week follow‐up period. We compared the mean daily symptom severity scores (0–3: none, mild, moderate, and severe) and change in symptoms between males and females using a multivariable linear mixed effects regression model.

**Results:**

The analysis included 223 enrolled adults with SARS‐CoV‐2 infection (58% females, mostly white, non‐Hispanic) from 146 households with 2917 total daily symptom reports. The overall mean severity of total symptoms reported over the illness period was 1.04 and 0.90 for females and males, respectively. Mean systemic and respiratory scores were higher for females than for males (*p* < 0.001). In multivariable analyses, females reported more severe total and systemic symptoms during the illness period compared with males. However, no significant differences in reported respiratory symptoms were observed.

**Conclusions:**

Our findings indicate that among ambulatory adults with SARS‐CoV‐2 infections, females reported slightly higher symptom severity during their illness compared with males.

## 1 BACKGROUND

COVID‐19, a respiratory disease caused by SARS‐CoV‐2, can range from mild to very severe with short‐ or long‐term complications.^1^, ^2^ While increasing age and presence of certain medical conditions, such as obesity and chronic pulmonary disease, have been associated with severe COVID‐19 outcomes, such as hospitalization and death,^3^ there is a growing body of literature demonstrating differences in the susceptibility to infections, clinical manifestations and associated outcomes between males and females.^4^, ^5^, ^6^, ^7^, ^8^ These sex differences are complex and have been at least partially attributed to different immunological responses to infection, as well as the influence of anatomical, hormonal, social, behavioral, and other differences.^9^ Importantly, sex differences may also impact the responses to preventative interventions, such as vaccination.^8^, ^10^

During the ongoing COVID‐19 pandemic, several studies have demonstrated similar incidence of COVID‐19 in males and females, while severe outcomes and deaths have been more commonly observed in males than in females.^11^, ^12^, ^13^, ^14^, ^15^ However, the majority of individuals with COVID‐19 have not needed hospital care, and the natural course of illness experienced by ambulatory patients with milder COVID‐19 in the community is not well understood. We used data from a prospective case‐ascertained household study to examine and compare reports of the severity and trajectory of symptoms between males and females with incident COVID‐19.

## 2 METHODS

### 2.1 Study design and population

This study used data from the COVID‐19—Influenza Transmission Evaluation Study (FluTES‐C), a prospective case‐ascertained study conducted in Nashville, Tennessee, which collected data between April 2020 and April 2021. Recruitment occurred within the integrated network of Vanderbilt University Medical Center (VUMC) walk‐in clinics dispersed throughout Davidson County, Tennessee, and surrounding areas. At these clinics, patients of any age who presented with acute respiratory symptoms were routinely tested for SARS‐CoV‐2 using real‐time reverse transcription polymerase chain reaction (RT‐PCR). The results of these clinical tests were generally available within 2 days of specimen collection. An index case was the first household member testing positive for SARS‐CoV‐2 who had the onset of symptoms in the 7 days before enrollment and lived with at least one other person who was not symptomatic at the time of the index case's illness onset. Index cases were identified and invited to participate, together with their household contacts. After providing consent and enrolling, the index case and household contacts answered questionnaires pertaining to sociodemographic data and past medical history. Data were collected using Research Electronic Data Capture (REDCap).^16^

After enrollment, all household members were followed for 14 days. During follow‐up, participants provided daily self‐collected nasal swab specimens for identification of viral infections and a daily symptom diary. Nasal swab specimens were tested at a research laboratory for the identification of SARS‐CoV‐2 infections using RT‐PCR. Cycle threshold (CT) values for SARS‐CoV‐2 N1 and N2 targets were recorded for specimens with positive detections as a surrogate for viral loads.

This study was reviewed and approved by the VUMC Institutional Review Board, with the Center for Disease Control and Prevention's (CDC) reliance on all reviews and approvals. Activities were conducted consistent with applicable federal law and CDC policy (see 45 C.F.R. part 46; 21 C.F.R. part 56).

#### 2.1.1 Daily measurement of symptom severity

We collected information daily on the presence of symptoms and medications taken during follow‐up on a subset of adult index cases and household participants. Index cases and household participants aged ≥18 years who reported at least one compatible symptom on a given day completed the Influenza Intensity and Impact Questionnaire (FluiiQ),^17^ starting on the day of symptom onset and continuing through the end of the 14‐day study follow‐up period. Every follow‐up day, diaries were distributed electronically to study participants during the late afternoon, and participants were asked to report symptoms experienced and healthcare use, including medications used. The application of the FluiiQ measurements was restricted to adults as these were designed and validated for examination of the intensity of influenza disease in adults.^17^ It includes five domains: respiratory symptoms, systemic symptoms, impact on daily activities, impact on emotions, and impact on relationships. Each domain consists of three to seven items with a 4‐point Likert scale response option (0–3), where the higher the score indicated, the higher the severity of symptoms, from 0 (*none*), 1 (*mild*), 2 (*moderate*), to 3 (*severe*). As planned, this study focused on respiratory (cough, sore throat, and nasal congestion), systemic (headache, feeling feverish, body aches and pains, fatigue, neck pain, interrupted sleep, and loss of appetite), and total (combined respiratory and systemic) symptoms to examine the severity and trajectory of symptoms reported by patients during the course of the SARS‐CoV‐2 infection.

### 2.2 Statistical analysis

We included participants who had completed at least five of the potential 14 FluiiQ daily questionnaires, with at least one FluiiQ daily questionnaire assessed during the first 5 days of symptoms, and those with at least one positive SARS‐CoV‐2 respiratory specimen in follow‐up. If a daily FluiiQ was missing, we imputed values within the period of observation using the following approaches: (i) if the missing values occurred after a zero score, then the last observed value (0) was carried forward; (ii) if a single value was missing between two recorded values, then the score from the day before was carried forward; and (iii) if there was more than one consecutive missing value or the first value during follow‐up was missing, multiple imputation by chained equations (MICE) with five replications was applied.^18^, ^19^

A daily total FluiiQ score was calculated by summing the responses for each symptom (i.e., adding up to a value from 0 to 30 when 10 symptoms were assessed) and then scaling the summation to extend between 0 and 3 to be consistent with the interpretation of the individual scores.^17^, ^20^ For example, total summation scores of 0 were scaled to 0, scores of 1–10 to 1, scores of 11–20 to 2, and scores of 21–30 to 3. Additionally, we derived each subject's highest IIQ score daily throughout the 2‐week follow‐up period. The same process was applied separately for the respiratory and systemic symptom domains. Summary statistics for these measures were calculated overall and by sex.

Enrollment demographic and clinical variables were compared between sex groups using Wilcoxon rank sum tests and Pearson's tests of homogeneity for continuous and categorical variables, respectively. Additionally, differences in CT values for N1 and N2 targets were tested using quantile ANOVA with random effects to account for intra‐subject correlation. We described and compared the severity and trajectory of daily symptoms over time between females and males using multivariable linear mixed effects models.^20^ These analyses accounted for the repeated daily assessments conducted on each individual while acknowledging the aggregation of individuals at the household level through random nested effects. Specifically, the model included random intercepts by household and subject (nested as ordered). The mixed effects models were adjusted for age, daily use of fever‐reducing medications, whether the participant was an index case or household contact, and the days from the first symptomatic date. Separate models were conducted for each domain of symptoms: total, systemic, and respiratory. A planned sensitivity analysis was conducted using a complete case scenario without imputing missing values. A separate sensitivity analysis repeated the primary analysis, with further adjustment for the presence of comorbidities. Statistical analyses were conducted in R 4.1.2 along with mice and lme4 packages (R Foundation for Statistical Computing, Vienna, Austria) and Stata version 17 (StataCorp, College Station, TX).

## 3 RESULTS

### 3.1 Study population

There were 441 subjects age 18 years or older from 165 households in Nashville enrolled in the FluTES‐C study. We first excluded those who did not have a laboratory‐confirmed SARS‐CoV‐2 infection (*n* = 112). Of participants with confirmed SARS‐CoV‐2 infection, we excluded those without any FluiiQ symptom assessments (*n* = 90), those who had all IIQ with 0 scores (*n* = 10), those who did not have at least one completed assessment within the first 5 days of symptom onset (*n* = 2), and those with less than five total completed assessments (*n* = 4). After application of selection criteria, 223 participants from 146 households were included in the current study analyses, including 130 females and 93 males. Age, race, and ethnicity were similar between the sex groups, with the majority (78%) of participants in the 18–50‐year‐old age group identified as white, non‐Hispanic (81%). The distribution of comorbidities was similar between females and males. Additionally, 139 (62%) participants were index cases, with a similar distribution among females and males. Among specimens tested with positive results, the RT‐PCR median and lowest CT values for N1 and N2 targets were consistently higher among females than among males (Table 1).

**TABLE 1:** Characteristics of study participants by sex and both sexes combined, FluTES‐C—Nashville TN April 2020–April 2021.

	Females *n* = 130	Males *n* = 93	Combined *n* = 223	*p*‐value*
Age in years, median (IQR)	36.0 (26.0–47.0)	38.0 (27.0–50.0)	37.0 (27.0–48.0)	0.163^a^
Age in years, % (*n*)				0.58^b^
18–49	80% (104)	74% (69)	78% (173)	
50–64	14% (18)	17% (16)	15% (34)	
≥65	6% (8)	9% (8)	7% (16)	
Race and ethnicity				0.945^b^
Hispanic White	8% (11)	6% (6)	8% (17)	
Hispanic Black	0% (0)	0% (0)	0% (0)	
Hispanic other	5% (6)	4% (4)	4% (10)	
Non‐Hispanic White	81% (105)	81% (75)	81% (180)	
Non‐Hispanic Black	5% (6)	6% (6)	5% (12)	
Non‐Hispanic other	2% (2)	2% (2)	2% (4)	
Index cases	65% (85)	58% (54)	62% (139)	0.266^b^
Fever medication use				0.079^b^
Did not take fever medication or unknown	8% (11)	16% (15)	12% (26)	
Took fever medication at least once	92% (119)	84% (78)	88% (197)	
CT values N1, median (IQR)	32.94 (29.03–35.93)	31.18 (27.56–34.68)	32.18 (28.28–35.51)	0.04^c^
CT values N2, median (IQR)	33.62 (29.30–36.61)	31.69 (27.73–35.16)	32.77 (28.57–36.13)	0.001^c^
Lowest CT values N1, median (IQR)	26.95 (24.24–31.61)	26.43 (23.94–29.80)	26.55 (24.00–30.46)	0.316^a^
Lowest CT values N2, median (IQR)	27.43 (24.36–32.24)	26.61 (23.71–30.09)	26.95 (23.81–30.95)	0.331^a^
Any comorbidities	35% (45)	26% (24)	31% (69)	0.161^b^
Asthma	15% (19)	10% (9)	13% (28)	0.285^b^
Cardiovascular/heart disease	3% (4)	3% (3)	3% (7)	0.950^b^
Diabetes	5% (6)	4% (4)	4% (10)	0.911^b^
Cancer	3% (4)	2% (2)	3% (6)	0.673^b^
Immunocompromised	2% (3)	1% (1)	2% (4)	0.494^b^
Extreme obesity	4% (5)	2% (2)	3% (7)	0.491^b^
Kidney disease	1% (1)	0% (0)	0% (1)	0.397^b^
Other pre‐existing conditions	11% (14)	12% (11)	11% (25)	0.805^b^

*Note*: Numbers in parentheses after proportions are frequencies, unless otherwise specified.

Abbreviations: CT, cycle threshold; IQR, interquartile range; SD, standard deviation.

^a^ Wilcoxon test.

^b^ Pearson test.

^c^ Quantile ANOVA with random effects based on 1228 positive tests.

* *p*‐values reflect comparisons between males and females.

### 3.2 Symptom measurements

The 223 study participants contributed 2917 daily symptom assessments. The number of symptom assessments collected per day was relatively stable throughout the first 10 days of follow‐up (range: 174–214 per day), with the lowest number of observations available on Day 14 (*n* = 174), the last day of the protocol‐specific observation period. There were a total of 92 symptom assessments missing among 32 participants, and the daily proportion of missing symptom assessments ranged from 0.9% to 4.2% and did not follow a specific pattern. These missing assessments were imputed for subsequent analyses (Table S1).

### 3.3 Severity of symptoms summarized during follow‐up overall and by sex

**FIGURE 1:**
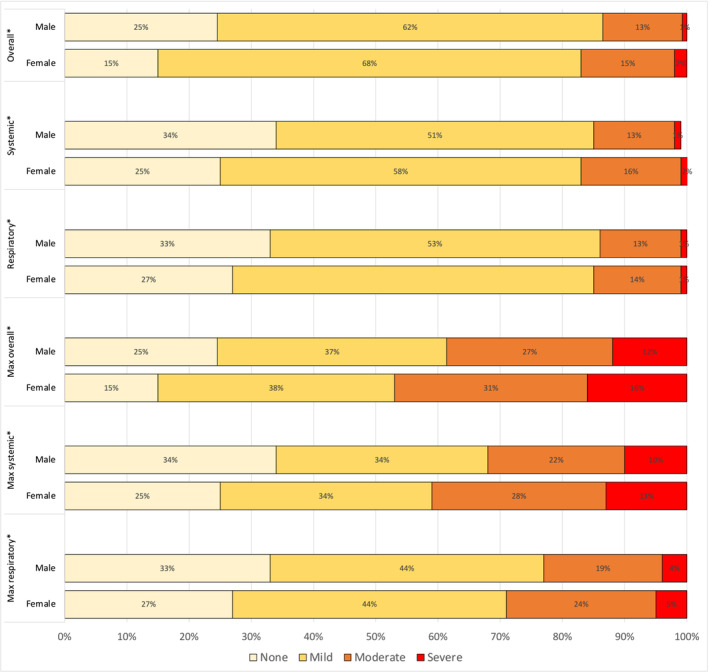
Distribution of mean daily total, systemic, and respiratory symptom scores and maximum scores during follow‐up by sex, FluTES‐C—Nashville TN April 2020–April 2021. *Indicates a statistically significant difference in proportions between sex groups.

The total mean symptom severity score computed during the follow‐up period indicated that 65% of study participants with confirmed SARS‐CoV‐2 infection reported mild symptoms, 14% moderate symptoms, and 1% severe symptoms. This score distribution, indicating that the majority of participants reported mild symptoms, was also consistently observed for both systemic and respiratory symptom domain scores (Figure 1). The mean total symptom score and its standard deviation during follow‐up was 0.98 ± 0.62, the mean systemic score was 0.90 ± 0.70, and the mean respiratory score was 0.86 ± 0.67. Females had consistently higher total, systemic, and respiratory scores than males; absolute scores differed by 0.14, 0.13, and 0.08 points, respectively. The mean maximum total, systemic, and respiratory scores were all higher for females than for males (*p* < 0.01) (Table 2).

**TABLE 2:** Mean and maximum symptom scores from the Influenza Intensity and Impact Questionnaire (FluiiQ) assessment conducted during follow‐up, FluTES‐C—Nashville TN April 2020–April 2021.

Daily scores/mean (SD)	Females *n* = 1712	Males *n* = 1205	Combined *n* = 2917	*p*‐value*
Total score	1.04 (0.61)	0.90 (0.63)	0.98 (0.62)	<0.001
Systemic score	0.95 (0.70)	0.82 (0.70)	0.90 (0.70)	<0.001
Respiratory sore	0.89 (0.66)	0.81 (0.68)	0.86 (0.67)	0.002
Maximum scores/mean (SD)				
Total score	1.48 (0.93)	1.26 (0.96)	1.39 (0.95)	<0.001
Systemic score	1.30 (0.98)	1.08 (0.98)	1.21 (0.99)	<0.001
Respiratory sore	1.08 (0.85)	0.93 (0.82)	1.02 (0.84)	<0.001

Abbreviation: SD, standard deviation.

* ANOVA with random effects.

Differences in mean symptom scores between females and males during follow‐up varied according to specific symptoms (absolute score differences range: 0.006–0.235). Although the mean symptom scores for cough, sore throat, fever, body aches, fatigue, neck pain, and sleep problems were similar between females and males, females had significantly higher mean scores for headache, nasal congestion, and appetite problems (*p* < 0.02 for each symptom) (Figure S1).

### 3.4 Trajectory of daily symptoms during follow‐up overall and by sex

**FIGURE 2:**
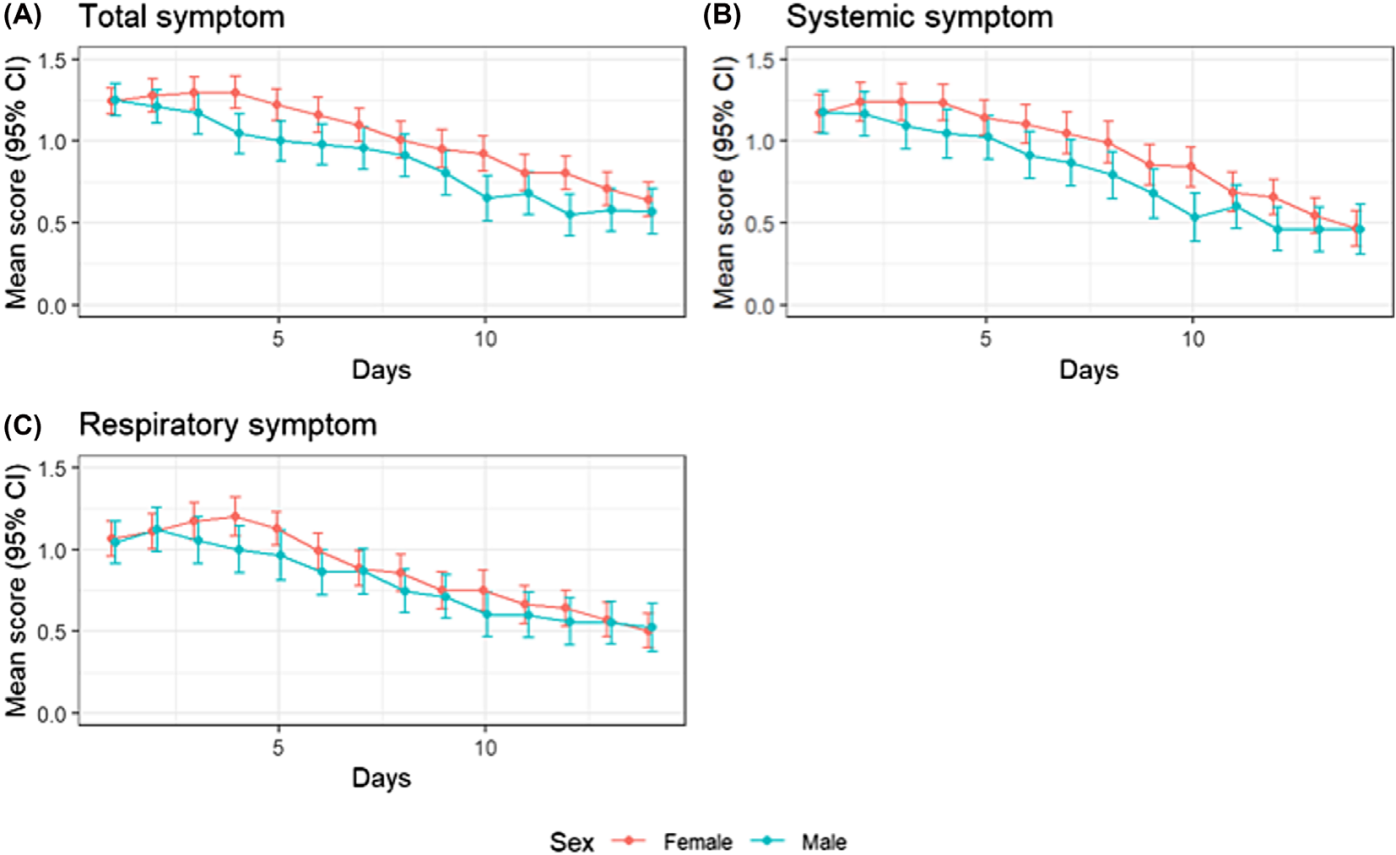
Trajectories of daily mean symptom scores* during follow‐up for total, systemic, and respiratory symptoms by sex, FluTES‐C—Nashville TN April 2020–April 2021. *For each data point in Figure 2, we computed 95% confidence intervals using mean scores and standard errors calculated for each combination of sex and day.

When examining the trajectories of daily symptoms since illness onset, the mean daily total, systemic, and respiratory systems were commonly reported higher in females than in males during the follow‐up period (Figure 2). While females and males started out with similar total, systemic, and respiratory scores, females on average continued to report higher symptom scores during the subsequent follow‐up period (Figure 2).

### 3.5 The role of sex on the severity and trajectory of daily symptoms

In the multivariable linear mixed effect models, females had higher total and systemic symptom scores than males (mean differences = 0.125; 95% CI: 0.036–0.214 and 0.105; 95% CI: 0.008–0.202, respectively). There were no significant differences in respiratory symptoms between sex groups. Similar differences were observed when maximum total, systemic, and respiratory scores were compared between sex groups. Sensitivity analyses restricted to observations with complete values and without data imputation (i.e., a complete case scenario) yielded virtually identical results to those from the primary analyses. Similarly, additional adjustment for comorbidities yielded similar results to those from the primary analyses (Table S2).

## 4 DISCUSSION

Several previous studies have evaluated sex differences among clinical outcomes for hospitalized or critically ill patients with COVID‐19, but few studies have characterized in detail the perception of severity and trajectory of reported symptoms in patients without severe disease. Understanding sex differences in the natural history of non‐severe COVID‐19 disease could have implications for public health measures and messaging as well as clinical and public health interventions. Thus, our findings build upon previous literature by examining the course of mild illness through detailed daily assessments of symptoms among subjects with laboratory‐confirmed COVID‐19. This prospective study showed that, among outpatients, adult females with COVID‐19, on average, reported greater severity of symptoms than males, although the severity of symptoms in both groups was generally mild.

Several studies have demonstrated biological differences between males and females with regards to susceptibility and course of infections.^4^, ^5^, ^9^ These differences are complex and may impact males or females differently depending on the infectious disease process.^9^ Furthermore, in addition to physiological effects on sex‐related clinical features, factors defined by social or cultural norms, including education, occupation, social roles, and healthcare seeking behaviors may play a role in sex differences in disease phenotype and symptoms reporting.^4^, ^5^, ^21^, ^22^ Consequently, there is an established interest in understanding the role of sex differences in infectious diseases.^9^ Studies examining differences in the infectious process can focus on many points along the disease continuum, including susceptibility to infections upon exposure to the virus, severity of disease, and occurrence of severe outcomes. The majority of previous studies examining sex differences in SARS‐CoV‐2 infection and COVID‐19 have focused on hospitalized patients and found generally higher severity and mortality among male than female patients with COVID‐19.^11^, ^12^, ^13^, ^14^, ^15^ However, few studies have evaluated potential sex differences by examining symptoms on a daily basis during the natural history of acute infection.

Prior research has reported underlying biological sex differences in immunological responses and hormonal modulation of gene expression of receptor enzymes and in immune responses to viral infections.^4^ In general, females develop stronger innate and adaptive immune responses against most viral infections and vaccines compared with males.^6^, ^7^ Among healthy individuals, males, compared with females, have lower CD3+ and CD4+ T cell counts, CD4+ to CD8+ cell ratios, and Th1 responses, and females exhibit higher cytotoxic T cell activity along with upregulated expression of antiviral, proinflammatory, and anti‐inflammatory genes, many of which have estrogen response elements in their promoters.^4^, ^5^, ^23^ In line with previous research on differences in vaccine reactogenicity and immunogenicity by sex, studies have also found that females have a greater reactogenicity to COVID‐19 vaccines, reporting more adverse symptoms or events compared with their male counterparts.^23^, ^24^ The more rapid and robust immune responses mounted by females could be contributing to the differences in symptoms demonstrated in this study. In contrast, male hormones (i.e., androgens) may promote inflammation and could further contribute to the more severe COVID‐19 complications seen in males, including blood clots, heart attacks, and strokes.^25^ These observations could also have long‐term implications. Some recent studies suggest that the same sex differences previously described could render females more susceptible to long‐term COVID syndrome and associated prolonged auto‐immune‐related diseases.^26^, ^27^

Furthermore, in addition to underlying biological differences, assessments of symptoms severity may be affected by differential symptoms reporting. Differences in symptoms reporting between females and males can be understood as differences in either the perception of symptoms, the experience of symptoms, or both. Some, but not all, studies have described that females are more likely to report somatic symptoms than males across a range of illnesses.^21^, ^22^, ^28^ Several factors may contribute to observed differences in reporting symptoms, including innate differences in symptom perception, potential differences in pain thresholds,^29^, ^30^ the influence of psychological conditions and chronic diseases, the socialization process, and gender bias in research and clinical practice.^21^, ^28^

Our findings should be interpreted in light of several limitations. The absolute difference in symptom severity observed in this study by sex was small, and the intensity of the symptoms reported by both males and females was mild. The study was observational, and the differences in symptoms by sex may be confounded or mediated in a way that was not explored in our study. However, this study provides a comprehensive assessment of the severity and trajectory of symptoms during COVID‐19 among ambulatory patients, complementing previous cross‐sectional studies and other assessments restricted to hospitalized patients. The FluiiQ questionnaire for the daily assessment of symptoms has been previously validated for the assessment of influenza disease severity but not for COVID‐19. Moreover, FluiiQ does not capture shortness of breath, chest pain, wheezing, abdominal pain, vomiting, diarrhea, or symptoms commonly reported in COVID‐19, such as loss of taste or smell. However, there are enough symptoms of COVID‐19 that are shared with influenza and assessed in the FluiiQ for the questionnaire to be an informative tool in examining sex differences in symptom severity. Additionally, study patients were identified in the household ambulatory setting, and potential participants with the most severe symptoms may not have been willing or too sick to participate in the study, thereby limiting the generalizability of our observations. Furthermore, the study was conducted during the initial year of the pandemic when the circulation of other respiratory viruses like influenza was unusually low and prior to the emergence of SARS‐CoV‐2 variants of concern, the frequent occurrence of reinfections, and the widespread availability of SARS‐CoV‐2 vaccines. On the one hand, it is a strength that we are describing the natural history of COVID‐19 in a mostly immune‐naïve study population; however, additional research will be needed to assess whether the observed sex differences are impacted by new viral variants, vaccinations, and re‐infections. Importantly, while biological sex differences may have influenced the trajectory of infections, viral detections among females had consistently higher RT‐PCR CT values (therefore lower viral loads) than among males, suggesting that viral loads could not explain the observed differences. Finally, our study participants were mostly young and of white race from middle Tennessee, and our findings may not be directly generalizable to other populations.

In summary, in this prospective study of household members with laboratory‐confirmed SARS‐CoV‐2 infection and with daily standardized ascertainment of symptoms, females reported slightly higher symptom severity than males throughout their illness. Understanding sex differences in COVID‐19 symptom patterns has implications for fashioning risk stratification tools, designing prophylactic and therapeutic interventions such as dosing schedules, understanding COVID‐19 epidemiology, and developing public health guidelines.

## AUTHOR CONTRIBUTIONS

Carlos G. Grijalva, Melissa A. Rolfes, and H. Keipp Talbot conceptualized the study. Carlos G. Grijalva, Natasha Halasa, and H. Keipp Talbot acquired funding for the study. All authors contributed to the development of data collection forms. Carlos G. Grijalva and H. Keipp Talbot led patient enrollment and data and sample collection. Carlos G. Grijalva, Alexandra M. Mellis, Melissa A. Rolfes, James Chappell, Carrie Reed, Jessica E. Deyoe, Yuwei Zhu, Ahra Kim, and H. Keipp Talbot supervised data and biological sample collection and analyses. Yuwei Zhu, Ahra Kim, and Trey McGonigle conducted data analyses. Samuel P. Massion and Amanda C. Howa prepared the first manuscript draft. All authors critically revised, edited, and approved the manuscript.

## CONFLICT OF INTEREST STATEMENT

C.G.G. reports grants from Syneos Health, the National Institutes of Health, the Food and Drug Administration, the Agency for Health Care Research and Quality, and consultation fees from Merck. The other authors have no potential conflicts of interest to report.

### PEER REVIEW

The peer review history for this article is available at https://www.webofscience.com/api/gateway/wos/peer-review/10.1111/irv.13235.

## Supporting information


**Figure S1.** Distribution of mean daily specific symptom scores during follow‐up by sex, FluTES‐C ‐ Nashville TN April 2020 – April 2021.
*Indicates statistically significant difference in proportions between sex groups.

**Table S1.** Symptom measurements (observed and imputed) by days since illness onset, FluTES‐C ‐ Nashville TN April 2020 – April 2021.
**Table S2.** Summary of estimated regression coefficients (sex parameter) from multivariable linear mixed effects model with and without data imputation* and after additional adjustment for an indicator for comorbidities.
*Multivariable models adjusted for age, daily use of fever‐reducing medications, whether the participant was an index or a household contact, and the days from first symptomatic date. See main text for description of missing data and imputation processes.

## Data Availability

The data are not publicly available due to ethical reasons. The data presented in this study are available on reasonable request to the corresponding author.
