# Supplementary figures and images for: Sex differences in COVID‐19 symptom severity and trajectories among ambulatory adults
Source: Influenza Other Respir Viruses. 2023 Dec 19;17(12):e13235. doi: 10.1111/irv.13235 (PMC10730332; doi:10.1111/irv.13235)

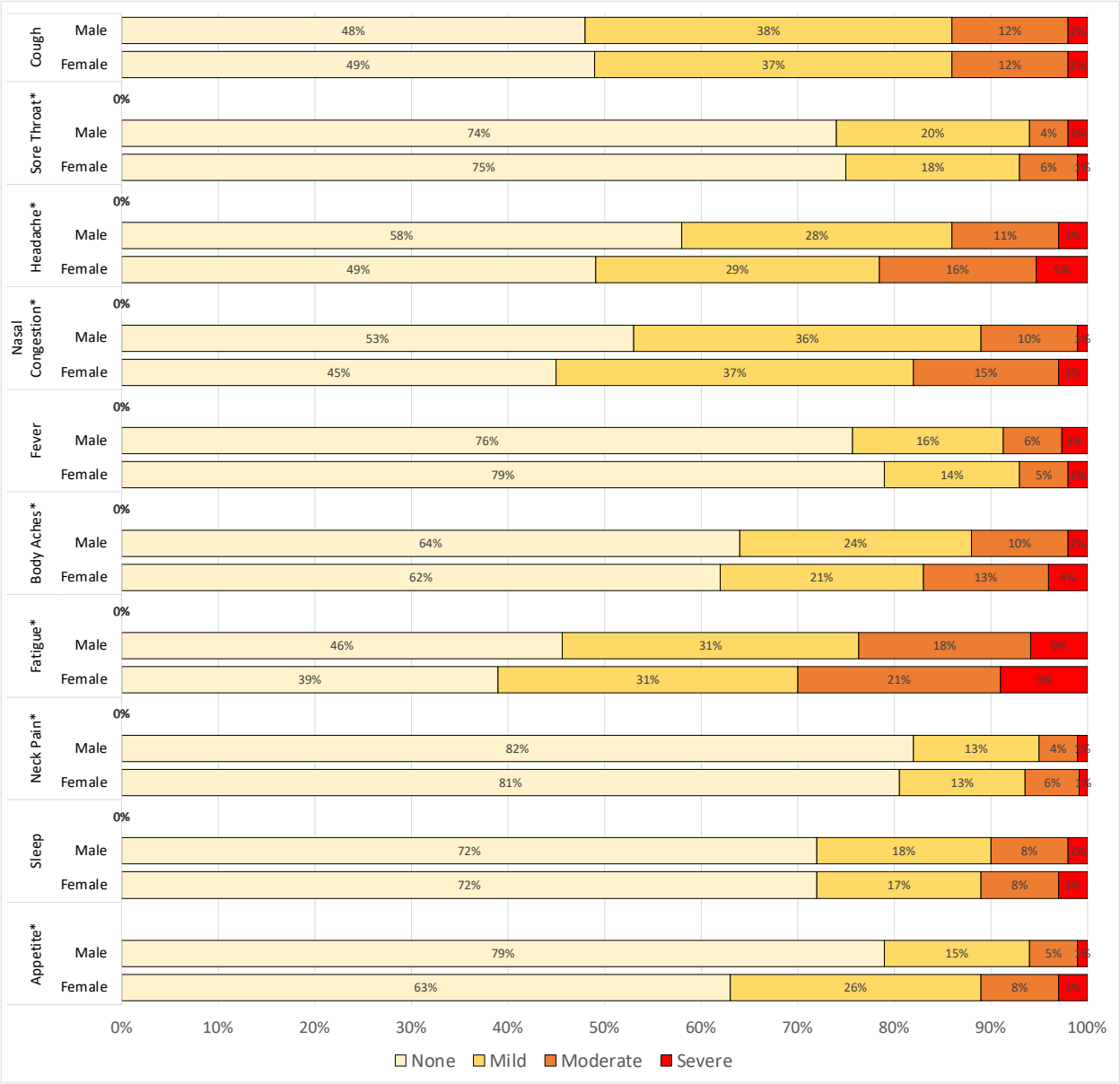

Supplement: Supplementary file 1 — Figure S1. Distribution of mean daily specific symptom scores during follow‐up by sex, FluTES‐C ‐ Nashville TN April 2020 – April 2021. *Indicates statistically significant difference in proportions between sex groups. [file IRV-17-e13235-s002.pdf]
