# Supplementary material for: Sex differences in COVID‐19 symptom severity and trajectories among ambulatory adults
Source: Influenza Other Respir Viruses. 2023 Dec 19;17(12):e13235. doi: 10.1111/irv.13235 (PMC10730332; doi:10.1111/irv.13235)
Supplement: Supplementary file 2 — Table S1. Symptom measurements (observed and imputed) by days since illness onset, FluTES‐C ‐ Nashville TN April 2020 – April 2021. Table S2. Summary of estimated regression coefficients (sex parameter) from multivariable linear mixed effects model with and without data imputation* and after additional adjustment for an indicator for comorbidities. *Multivariable models adjusted for age, daily use of fever‐reducing medications, whether the participant was an index or a household contact, and the days from first symptomatic date. See main text for description of missing data and imputation processes. [file IRV-17-e13235-s001.docx]

**Supplementary Table 1**. Symptom measurements by days since illness onset, FluTES-C - Nashville TN April 2020 – April 2021

| Day | Number OF Symptom Assessments (%) | number of imputed symptom assessments (%) | Total Symptom assessments (including imputed records) |
| --- | --- | --- | --- |
| 1 | 214 (96.0%) | 9 (4.0%) | 223 |
| 2 | 216 (96.9%) | 7 (3.1%) | 223 |
| 3 | 215 (96.4%) | 8 (3.6%) | 223 |
| 4 | 218 (97.8%) | 5 (2.2%) | 223 |
| 5 | 217 (97.3%) | 6 (2.7%) | 223 |
| 6 | 220 (98.1%) | 2 (0.9%) | 222 |
| 7 | 214 (96.4%) | 8 (3.6%) | 222 |
| 8 | 216 (98.2%) | 4 (1.8%) | 220 |
| 9 | 211 (96.8%) | 7 (3.2%) | 218 |
| 10 | 206 (95.8%) | 9 (4.2%) | 215 |
| 11 | 204 (96.2%) | 8 (3.8%) | 212 |
| 12 | 199 (96.1%) | 8 (3.9%) | 207 |
| 13 | 193 (96.5%) | 7 (3.5%) | 200 |
| 14 | 174 (97.8%) | 4 (2.2%) | 178 |
| Total | 2917 | 92 | 3009 |

**Supplementary Table 2.** Summary of estimated regression coefficients (sex parameter) from multivariable linear mixed effects model with and without data imputation* and including adjustment for comorbidities

| Analysis | Outcome | Difference in score  (male vs. female) | Lower 95% Confidence Limit | Upper 95% Confidence Limit |
| --- | --- | --- | --- | --- |
| Primary analyses (with data imputation) | Mean Overall Score | -0.1251 | -0.2142 | -0.0360 |
|  | Mean Systemic Score | -0.1050 | -0.2024 | -0.0076 |
|  | Mean Respiratory Score | -0.0617 | -0.1624 | 0.0390 |
|  | Maximum Overall Score | -0.1859 | -0.3174 | -0.0544 |
|  | Maximum Systemic Score | -0.1777 | -0.3078 | -0.0476 |
|  | Maximum Respiratory Score | -0.1183 | -0.2466 | 0.0101 |
| Sensitivity analyses (complete case – without data imputation) | Mean Overall Score | -0.1270 | -0.2170 | -0.0375 |
|  | Mean Systemic Score | -0.1021 | -0.2010 | -0.0043 |
|  | Mean Respiratory Score | -0.0611 | -0.1635 | 0.0418 |
|  | Maximum Overall Score | -0.1853 | -0.3189 | -0.0519 |
|  | Maximum Systemic Score | -0.1755 | -0.3056 | -0.0460 |
|  | Maximum Respiratory Score | -0.1195 | -0.2507 | 0.0121 |
| Primary analyses (with data imputation) including adjustment for comorbidities | Mean Overall Score | -0.1193 | -0.2094 | -0.0297 |
|  | Mean Systemic Score | -0.0915 | -0.1901 | 0.006 |
|  | Mean Respiratory Score | -0.0574 | -0.1605 | 0.0459 |
|  | Maximum Overall Score | -0.1761 | -0.3103 | -0.042 |
|  | Maximum Systemic Score | -0.165 | -0.2954 | -0.0352 |
|  | Maximum Respiratory Score | -0.114 | -0.246 | 0.0183 |

*Multivariable models adjusted for age, daily use of fever-reducing medications, whether the participant was an index or a household contact, and the days from first symptomatic date. See main text for description of missing data and imputation processes
